# Autotaxin–Lysophosphatidic Acid Signaling in Alzheimer’s Disease

**DOI:** 10.3390/ijms19071827

**Published:** 2018-06-21

**Authors:** Sindhu Ramesh, Manoj Govindarajulu, Vishnu Suppiramaniam, Timothy Moore, Muralikrishnan Dhanasekaran

**Affiliations:** Department of Drug Discovery and Development, Harrison School of Pharmacy, Auburn University, Auburn, AL 36849, USA; szr0065@auburn.edu (S.R.); myg0003@auburn.edu (M.G.); suppivd@auburn.edu (V.S.)

**Keywords:** autotaxin, lysophosphatidic acid, GPCR, Alzheimer’s disease

## Abstract

The brain contains various forms of lipids that are important for maintaining its structural integrity and regulating various signaling cascades. Autotaxin (ATX) is an ecto-nucleotide pyrophosphatase/phosphodiesterase-2 enzyme that hydrolyzes extracellular lysophospholipids into the lipid mediator lysophosphatidic acid (LPA). LPA is a major bioactive lipid which acts through G protein-coupled receptors (GPCRs) and plays an important role in mediating cellular signaling processes. The majority of synthesized LPA is derived from membrane phospholipids through the action of the secreted enzyme ATX. Both ATX and LPA are highly expressed in the central nervous system. Dysfunctional expression and activity of ATX with associated changes in LPA signaling have recently been implicated in the pathogenesis of Alzheimer’s disease (AD). This review focuses on the current understanding of LPA signaling, with emphasis on the importance of the autotaxin–lysophosphatidic acid (ATX–LPA) pathway and its alterations in AD and a brief note on future therapeutic applications based on ATX–LPA signaling.

## 1. Introduction

Alzheimer’s disease (AD) is a chronic neurodegenerative disorder characterized by cognitive impairment and behavioral abnormalities. The incidence of AD is increasing annually, especially in the western world [1]. Despite continued research, there is presently no cure or treatment for AD. Conventional therapies provide only symptomatic relief and have limited influence on the natural course of the disease. However, in recent years, significant efforts aimed at identifying novel molecular mechanisms have advanced the understanding of the disease pathogenesis. One such mechanism that has received extensive consideration is the autotaxin–lysophosphatidic acid (ATX–LPA) axis, as increasing evidence indicates that ATX–LPA signaling plays a crucial role in numerous cellular processes [2]. Additionally, various research studies and clinical trials are underway to study the therapeutic potential of ATX inhibitors and LPA receptor antagonists. This review provides a brief update on the activities of ATX and LPA, with emphasis on their physiological functions, regulation, and emerging role in the pathophysiology of neurodegenerative diseases like AD.

## 2. Autotaxin (ATX)

Autotaxin (ATX) is an ecto-nucleotide pyrophosphatase/phosphodiesterase enzyme (ENPP), encoded by the *ENPP2* gene. To date, the *ENPP* gene family consists of seven members (*ENPP1* to *ENPP7*) numbered according to their order of discovery. The enzymes encoded by the *ENPP* gene family are structurally related to each other and mediate the hydrolysis of the phosphodiester bonds of nucleoside triphosphates, choline phosphate esters, and lysophospholipids [3,4]. ATX is important in converting lysophospholipids such as lysophosphatidylcholine (LPC), lysophosphatidylethanolamine, and lysophosphatidylserine into lysophosphatidic acid (LPA) [5,6,7,8,9]. ATX is widely expressed, with the highest mRNA levels identified in the brain, spinal cord, ovary, lung, intestine, kidney, and lymph nodes [10,11]. Interestingly, the adipose tissue and endothelial cells in the lymphatics secrete high ATX levels, and the adipose specific deletion of ATX leads to 90% reduction in plasma ATX [12,13].

Structurally, ATX consists of two N-terminal Cys-rich somatomedin B-like (SMB) domains, a central catalytic phosphodiesterase (PDE) domain, and a C-terminal nuclease-like (NUC) domain that is catalytically inactive. The SMB domain is involved in protein–protein interactions, the central catalytic side is essential for hydrolyzing lysophospholipids, and the NUC domain binds to calcium via an EF-hand-like motif, the precise function of which remains, however, to be determined [14,15].

ATX is synthesized as a pre-pro-enzyme and undergoes posttranslational modification (*N*-glycosylation) followed by proteolytic maturation before being secreted as an active lysophospholipase D (lysoPLD) [16,17,18]. The secreted ATX acts more locally rather than systemically, and the circulating ATX is responsible for maintaining adequate plasma LPA levels. The *ATX* gene undergoes alternative splicing to produce various isoforms (α, β, γ, and δ) [19]. All the isoforms possess similar lysoPLD activities and substrate preferences. However, the specific functions of each isoform are yet to be determined. The most predominant isoform, ATXβ, accounts for the majority of LPA production in the circulation. This circulating ATX has a short half-life due to its rapid degradation in the liver [20]. 

However, all the biological functions mediated by ATX are due to LPA production followed by its action on G protein-coupled receptors (GPCRs) [7]. Regardless of the extensive advancement in understanding ATX–LPA receptor signaling, the internal workings of ATX have long remained obscure. Structural studies of ATX have provided new insights into its function, thereby substantiating ATX as a unique lysoPLD [15].

## 3. Lysophosphatidic Acid and its Receptors

Lysophospholipids are components of the membrane that possess a large polar head and a single hydrophobic carbon chain. LPA belongs to a group of lyso-type glycerolipids having a glycerol backbone with an ester-linked fatty acid chain (acyl chain in position 1 or 2) and a polar phosphate group [21,22]. Hence, LPA refers to 1-acyl-2-hydroxy-*sn*-glycero-3-phosphate, but other forms such as 1-alkyl- or 2-acyl-LPA also exist [23,24]. In humans, the most abundant forms of LPA are 16:0-LPA (consisting of a palmityl chain), followed by 18:2-LPA, and 18:1-LPA [25]. The 18:1-LPA form is the most common laboratory agent used for signaling studies. They act as extracellular signaling mediators by acting through GPCRs and are involved in a broad range of biological process. LPA is widely distributed intracellularly and extracellularly in various tissues and organ systems. In the nervous system, it is predominantly expressed in the neural progenitor cells, astrocytes, microglia, and oligodendrocytes [26,27]. The biological fluids such as plasma, saliva, tears, aqueous humour, and cerebrospinal fluid contain biologically significant concentrations of LPA [23,28]. The identification and quantification of either total LPA or specific forms of LPA in a sample can be done using various methods, including calorimetric assays and mass spectrometry [29].

There are at least four pathways involved in LPA production, of which the autotaxin pathway is the most significant. The major pathway involves the enzyme ATX, which cleaves LPC to form LPA and choline [19]. Other lysophospholipids (lysophosphatidylserine and lysophosphatidylethanolamine) are also enzymatically processed to produce LPA. The other pathways leading to the formation of LPA include the hydrolysis of phosphatidic acid (PA) by membrane-bound phospholipase A1α or β [30]. The LPA formed extracellularly binds to the LPA receptor and acts as a signaling substance. Conversely, LPA can also be formed intracellularly from the mitochondria and the endoplasmic reticulum and acts as a substrate for glycerolipid synthesis. This is mediated by the conversion of glycerol 3-phosphate by glycerol 3-phosphae kinase and the conversion of monoacylglycerol by monoacylglycerol kinase [30]. It is unsure whether the intracellularly produced LPA can cross the plasma membrane into the extracellular compartment and act as signaling molecule.

The first reported in vivo biological function attributed to LPA was blood pressure regulation [31]. Later, LPA was found to stimulate cell proliferation in a pertussis toxin-sensitive manner, which suggested that LPA acts through its cognate GPCR [32]. Various gain- and loss-of-function studies have elucidated a wide range of cellular functions mediated by LPA, which include cell proliferation, cell migration, cell survival, and cytoskeletal reorganization [33,34]. These LPA-mediated physiological and pathological processes involve nervous system and vascular development, reproduction, wound healing, immune system function, inflammation, cancer, angiogenesis, and obesity [35,36,37,38,39,40]. The diverse and numerous physiological effects of LPA are mediated through six currently recognized LPA receptors (LPARs), namely, LPA1–LPA6. The gene names are *LPAR1–LPAR6* in humans and *Lpar1–Lpar6* in murine models and non-human species [41]. The binding of LPA to its GPCRs leads to the activation of Gα subunits, namely, G12/13, Gq/11, Gi/o, and Gs, which leads to downstream signaling cascades [42,43] and various cellular effects, as described in Table 1.

Each LPAR has multiple important regulatory functions throughout the body [23]. Many of these have been elucidated using knockout animals, pharmacological LPAR agonists or antagonists, and gene association studies. The various subtypes of the LPARs and their physiological functions are illustrated in Table 2.

### LPA Receptors in the Central Nervous System

Almost all types of LPA receptors are present in the brain and show varied expression depending on the anatomical location and the neuronal cell type. 

Neural progenitor cells (NPCs) from the developing mouse cerebral cortex mediate neurogenesis by differentiating into a restricted set of neuronal and glial cell types. LPA signaling regulates NPC-mediated neurogenesis through three subtypes of LPA receptors: LPA1, LPA2, and LPA4 [62]. The role of LPA1 in cortical development was established through studies utilizing LPA1-deficient NPCs or whole cell cultures [63,64]. The neurogenesis-related responses induced by LPA are lost in NPCs from LPA1-null mice, indicating that LPA1 plays an important role in modulating neurogenesis. Additionally, studies performed on organotypic whole cortex showed that LPA-induced differentiation of NPCs was mediated by LPA1 and partly by LPA2 [64]. In NPCs, LPA causes neurotransmitter-like stimuli of ionic conductance, thereby indicating that LPA is an important physiological factor in cortical development [65]. With the current information illustrating the significance of LPA signaling in neurogenesis, further investigation is required to elucidate the mechanisms and probable roles of other LPA receptors. 

During neurogenesis, LPA, acting through LPA1 and the Gi/o pathway, promotes cortical NPCs to form the neuronal lineage. In neurons, LPA also mediates actin cytoskeleton and microtubules formation, as well as morphology and motility of newly formed postmitotic neurons [27,66]. Sprouting, extension, and branching of neurites are important for neuronal network formation during development and involve cytoskeletal rearrangements. All LPA receptor subtypes induce neurite retraction and growth cone collapse, except for LPA3 [67]. The neurite retraction response is mediated by LPA acting through the Rho-associated protein kinase (ROCK) pathway, which is discussed in the following sections. The activation of LPA3 has been shown to induce axonal branching or neurite branching in hippocampal cell cultures; this response is mediated through Gq and the Rho family GTPase 2 (Rnd2) family [68]. Newly formed neurons establish connections through the specification and development of neurites into axons and dendrites. Axonal sprouting has been found to be influenced by LPA-mediated signaling [69]. LPA signaling is also important in mediating synapse formation and transmission, cell death/survival, and neuronal migration [70,71,72]. LPA receptors, especially LPA1, are important in mediating synaptic plasticity and learning. Transfection of cultured hippocampal neurons with LPA1 caused a marked increase in dendritic spine density and size. In addition to its effects on morphology, LPA induced chloride currents in cortical neuroblasts and thereby activated dopamine release in PC12 cells. Consequently, LPA is thought to function as a neuromodulator [73,74]. LPA1^−/−^ mice display neurophysiological alterations despite being viable. Thus, LPA1 mediates the endogenous LPA effects in cerebral cortical neuroblasts, which indicates that a loss of normal LPA-dependent proliferation in lpA1(^−/−^) cortex may in part be compensated for by increased proliferative responses to basic fibroblast growth factor [75]. The mechanisms of synaptic plasticity changes include protein kinase C (PKC) activation, with Rho- and Rac-dependent, actin-based, morphological changes [76,77]. Overexpression of LPA1 in primary hippocampal neurons increased the density and size of dendritic spines with no significant changes in the magnitude and rate of miniature excitatory synaptic currents (mEPSC). However, the decay time of mEPSC was enhanced, indicating that LPA1 induces morphological and functional properties of dendritic spine synapses [73]. One study demonstrated that LPA administration to cultured hippocampal neurons resulted in apoptosis and necrosis by persistently increasing the intracellular calcium levels [78]. Interestingly, LPA treatment at physiological doses of excitatory presynaptic neurons decreased the amplitude of post-synaptic neuron potential, leading to a phenomenon called short-term depression (STD) [79]. LPA colocalizes with markers of neurotransmitter-containing vesicles and stimulates myosin light-chain kinase (MLCK), thereby promoting actomyosin contraction. This inhibits the movement of vesicles to the membrane, thereby inhibiting neurotransmitter release. LPA-mediated actions on inhibitory neurons involve acting on the post-synaptic neuron; this is in contrast with its action on excitatory neurons, which involves the stimulation of pre-synaptic neurons. LPA colocalizes with markers of a protein involved in assembling gamma-aminobutyric acid (GABA) receptors and acting on the RhoA–ROCK pathway, causing dephosphorylation of GABA subunits. This leads to GABA receptors’ inactivation and reduced GABA signaling, leading to short-term depression. This LPA-induced STD is helpful in slowing down neurotransmission in case of excess signaling which can lead to excitotoxicity. This fine-tuning of the synaptic behavior over a short duration is important in maintaining neuronal and synaptic homeostasis [80]. An altered lipid metabolism, as seen in various metabolic diseases, can cause LPA dysfunction, leading to various metabolic and neurological diseases. Synaptic regulation, neurogenesis, and neuronal death/survival all play a crucial role in memory impairments, and LPA signaling is thought to regulate these responses. In addition, LPA induces chloride currents in cortical neuroblasts and activate dopamine release in neuronal cell cultures, thereby suggesting that LPA can function as a neuromodulator [81]. LPA6 is the most recently characterized LPAR, and mutations of this receptor in humans are associated with certain forms of hair loss [60]. However, the functions of LPA6 in the central nervous system (CNS) remain to be characterized. 

Astrocytes are the most abundant neuroglial cells in the CNS, known to mediate several intricate, essential functions and pathological processes. The expression patterns of LPARs in astrocytes are interesting in that only LPA1 is detected in vivo, while LPA1–5 are seen in cultured astrocytes [82,83,84]. However, LPA6 expression in astrocytes has not yet been investigated. Of all the LPARs, LPA1 has been shown to be involved in neuronal differentiation in the developing cerebral cortex and to influence astrogliosis [63,64]. LPA-mediated proliferation of astrocytes is mediated by LPA1, as indicated by the absence of proliferation in astrocytes lacking LPA1 [85]. LPA-primed astrocytes secrete several soluble factors which facilitate neuronal differentiation, axonal growth of neurons, and epidermal growth factor signaling, indicating the importance of LPA signaling in astrocytes [86,87]. LPA induces diverse cellular responses in cultured astrocytes by acting through LPA1–3 [88,89]. The effects of LPA on cultured astrocytes include intracellular calcium mobilization, reactive oxygen species formation, actin cytoskeletal rearrangement, and decreased uptake of glutamate and glucose, which can contribute to neurodegeneration [90,91]. LPA induces the expression of immediate-early genes and cytokine genes, such as nerve growth factor and interleukins IL-1b, IL3, and IL-6 [82]. Lipopolysaccharide (LPS) or IL-1β stimulate the astrocytes and induce LPA-responsive astrocyte migration [92]. The cross-talk between astrocytes and neurons is crucial for normal physiological functioning in the CNS and in the regenerative process following various diseases. LPA signaling in astrocytes might contribute to the regulation of regeneration processes through neuronal differentiation. Conversely, excess LPA generated through astrocyte responses, like glutamate uptake blockade and cell proliferation, can lead to neuronal death and neurodegeneration. Further research on the critical role of LPA signaling in astrocytes will aid in understanding the complex neuropathology of neurodegenerative diseases like AD and in developing therapeutics for these diseases. 

Microglia are immune cells of the CNS, which play a crucial role in maintaining homeostasis in the brain [93,94]. Microglia are involved in the regulation of inflammation and in repair, regeneration, cytotoxicity, and immunosuppression, depending on their different activation states [95]. Following neuronal injury, microglial activation is induced following the release of various substances, like cytokines, ATP, growth factors, and changes in ion equilibrium [96]. These cellular processes are mediated by LPA signaling acting through microglial activation. Various studies have reported that LPA binds to and modulates the glial cells in the CNS [26,97,98]. For instance, human and murine microglial cell lines have been shown to express LPA receptors [99,100]. LPA binding induces membrane hyperpolarization by the activation of Ca^2+^-dependent K^+^ channels leading to microglial migration [101,102]. Other actions of LPA include energy homeostasis [100], oxidative stress response [103], and pro-inflammatory cytokine production [104]. The LPA-dependent microglial responses are mediated by intracellular phosphorylation cascades through the activation of protein kinase D and its isoforms (PKD1–3), thereby leading to inflammatory responses [105,106]. The mechanisms of the inflammatory response mediated by PKD includes the activation of either nuclear factor kappa light chain enhancer of activated B cells (NF-kB) signaling or oxidative stress pathways [107,108]. Similarly, exogenous LPA treatment of microglial cells induced the activation of PKD, Mitogen-activated protein kinase (MAPK), and AKT pathways in addition to phosphorylating signal transducers and activators of transcription (STAT1, STAT3) and phosphorylating c-Jun. These signaling pathways are crucial in regulating microglial polarization and chemotaxis [109,110]. Finally, increased LPA levels promote a pro-inflammatory phenotype. A better understanding of LPA-mediated effects on the glial cells of the CNS is important for studying disease patterns and unravelling potentially new targets to modulate neuroinflammation. 

Oligodendrocytes are glial cells in the CNS, which play an important role in the myelination process. They are also crucial for CNS development and post-injury repair, which occurs through remyelination. Oligodendrocytes express LPA1 in a spatiotemporal manner that correlates with maturation and myelination [111]. Extracellular-regulated-kinase 1/2 (ERK1/2) phosphorylation, process retraction, and cell rounding in mature oligodendrocytes were noted on LPA treatment in an in vitro study. However, no changes were observed in oligodendrocyte precursors [112]. Lastly, process formation and increased number of differentiating but not mature oligodendrocytes were noted upon LPA administration [113]. 

The development of the vascular network in the CNS involves angiogenesis, maturation of the vessels, and blood–brain barrier (BBB) formation [114,115]. These biological responses are mediated by vascular endothelial growth factor (VEGF), platelet-derived growth factor (PDGF), and Notch signaling [115,116]. Similarly, LPA and its receptors play an important role in angiogenesis and vascular development [36]. The expression of VEGF is induced by LPA acting through NF-kB signaling. Additionally, ATX and LPA1 expression is enhanced by VEGF in endothelial cells [117,118,119]. LPA receptor subtypes individually affect vascular development and homeostasis. LPA1-null and LPA2-null mice are associated with frontal cerebral hematomas, while LPA4-null mice exhibit dilated blood vessels and lymphatics due to dysfunctional pericytes. An in vitro model of angiogenesis showed that the pericytes could degrade LPA, thereby stabilizing the blood vessels. Cultured endothelial cells (ECs) express all subtypes of LPARs. LPA acting through LPARs regulates survival, proliferation, and EC migration, thereby influencing the vascular tone. A study done by Tigyi et al., using a piglet model of intracranial hematoma, proved that LPA exposure produces dose-dependent vasoconstriction, which is similar to the vasoconstriction produced by hemorrhage [120]. Recently, evidence has emerged that identifies LPA as a mediator of vasodilation via LPA1, phospholipase C, and endothelial nitric oxide synthase [121]. LPA overexposure has been reported to increase BBB permeability, which can occur in various pathological diseases [122,123].

The major site of cerebrospinal fluid (CSF) production, the choroid plexus, consisting of epithelial cells surrounded by capillaries, also express LPARs. In humans, LPA6 is expressed in the lateral ventricular choroid plexus [124], whereas, in the mouse embryo, LPA5 expression is prominent in the fourth ventricular choroid plexus [125]. Significantly, increased expression of ATX is noted in the choroid plexus [126,127]. Since various neuronal structures are located near the choroid plexus, which expresses high levels of ATX, ATX in the choroid plexus might catalyze the activity of LPC present in the circulation, thus promoting the formation of bioactive LPA and affecting various aspects of neurodevelopment. 

## 4. Altered ATX–LPA Signaling and LPARs in Alzheimer’s Disease

### 4.1. ATX–LPA Signaling and Amyloid β

Several studies indicate that amyloid β (Aβ) deposition is an important process in the pathogenesis of AD [128]. Accumulation of Aβ initiates downstream events, including hyperphosphorylation of tau and synaptic failure which results in neuronal death [129,130]. In the amyloidogenic pathway, the amyloid precursor protein (APP) is sequentially cleaved by β-secretase and γ-secretase to produce the toxic Aβ peptide consisting of 39–43 amino acids in length. β-secretase, also known as β-site APP-cleaving enzyme 1 (BACE1), is the rate-limiting step in the production of Aβ [131]. Increased expression of APP, β- and/or γ-secretases would result in increased Aβ production. β-secretase is a type I membrane aspartyl protease [132,133], whereas γ-secretase is a complex composed of four subunits, namely, presenilin (PS1 or PS2), nicastrin, Aph-1, and Pen-2, of which presenilin is a putative catalytic subunit [134]. 

Recent research suggests that vascular factors additionally play a critical role in the pathogenesis of AD [135,136,137]. Amongst the various vascular factors, oxidized low-density lipoprotein (oxLDL) has been implicated in AD pathogenesis [138,139]. Decreased levels of antioxidant enzymes activities and increased malondialdehyde levels (a marker of lipid peroxidation) were observed in AD patients in comparison to healthy non-demented control subjects [135,140]. The human CSF consists of high levels of lipoproteins, which are vulnerable to oxidation in AD and are neurotoxic when oxidized [141]. A strong positive correlation between the CSF levels of Aβ and oxLDL has been observed in AD patients, indicating that oxLDL is a key contributor to Aβ production [142]. LPA, the major bioactive component of oxLDL, has been shown to disrupt the BBB function and lead to various pathologies in AD [143]. A study conducted by Shi et al., utilizing an in vitro mouse neuroblastoma N2a cell line [stably expressing wild-type presenilin 1 (PS1wt) and Swedish mutant APP (APPsw), showed that LPA increases the production of Aβ by upregulating the expression of β-secretase (BACE1) without altering the expression of APP or γ-secretase complex proteins [144]. 

**Figure 1:**
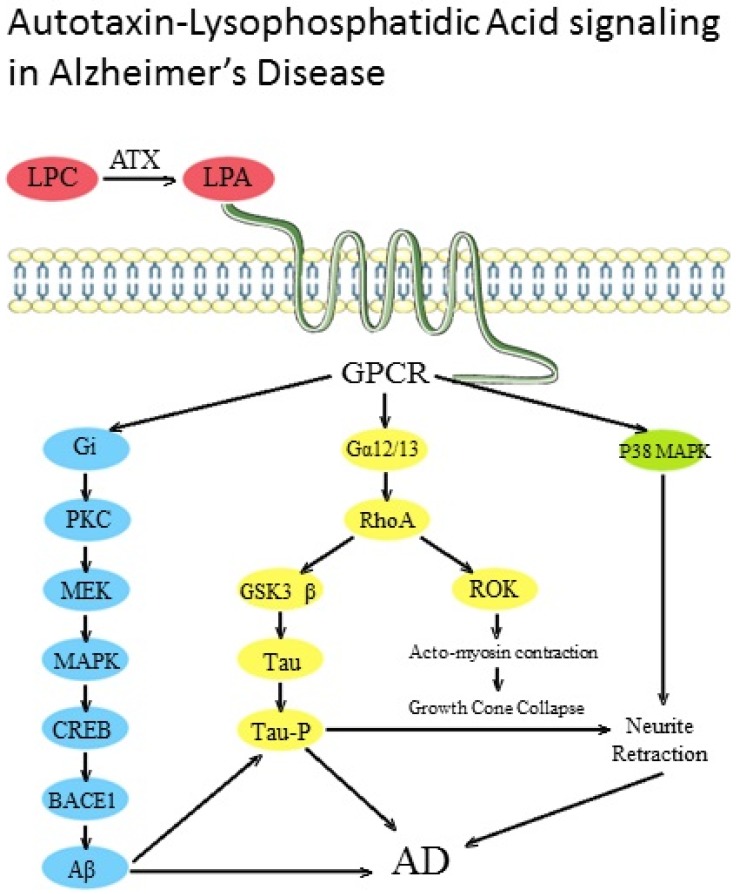
Autotaxin (ATX)–LPA-mediated downstream signaling leading to formation of amyloid-β (Aβ), hyperphosphorylation of tau, and neurite retraction all leading to the pathogenesis of Alzheimer’s disease (AD). GPCR, G protein-coupled receptors; PLC, Phospholipase C; PKC, Protein Kinase C; BACE1, β-Site APP-cleaving enzyme 1; CREB, cyclic AMP response element binding protein.

The upregulation of BACE1 expression by LPA occurs at the transcriptional level, as evident by the increased BACE1 mRNA levels. The promoter of the *BACE1* gene consists of the cAMP response element (CRE) and several other transcription factors binding sites. LPA significantly increases the binding activity of cAMP response element binding protein (CREB) to the CRE site of the BACE1 promoter without significantly altering the binding of other transcription factors. Hence, these results indicate that CREB mediates BACE1 upregulation in response to LPA. Protein kinase C (PKC) and its isoforms play an important role in regulating APP processing. For instance, the isoforms PKCα, PKCβ, and PKCε are implicated in regulating α-secretase-mediated APP processing [145,146,147]. In contrast, the PKCδ isoform mediates LPA-induced upregulation of β-secretase expression and Aβ production [148]. The LPA-mediated intracellular signaling cascade involves the activation (phosphorylation) of PKCδ, MEK, MAPK (Erk44/42), and p90RSK. Finally, p90RSk (a downstream target of PKC and MEK) is known to mediate CREB phosphorylation, thereby inducing CREB binding activity in response to the activation of upstream PKCδ [149]. Additionally, the two transcriptional factors CREB and ATF2 are phosphorylated, suggesting that CREB mediates the upregulation of BACE1 expression. Hence, LPA-induced BACE1 promoter activation is mediated by PKCδ–MEK–MAPK–p90RSk–CREB signaling cascades [144], as depicted in Figure 1.

Numerous studies demonstrate that cholesterol plays an important role in Aβ regulation; increased cholesterol levels are associated with increased Aβ production and accumulation [150]. Lipid rafts are membrane domains consisting predominantly of cholesterol and sphingolipids. They play an important role in mediating membrane trafficking, ligand binding, axonal development, and synaptic plasticity. Lipid rafts have been implicated in the pathogenesis of AD by enhancing the interaction between APP and BACE-1, thereby promoting the accumulation of Aβ peptide [151]. BACE1 regulation and its interaction with APP is lipid-dependent and involves lipid rafts [152]. Furthermore, oxLDL has been shown to increase lipid raft formation and the association of BACE1 activity with lipid rafts, thereby increasing Aβ levels. The postulated mechanism involves the depletion of intracellular glutathione due to oxidative stress, which leads to an increase in acid sphingomyelinase activity [153]. 

### 4.2. ATX–LPA Signaling and Tau Hyperphosphorylation

Tau (tubulin-associated unit) is a key protein essential for microtubule assembly and constitutes one of the most important microtubule-associated proteins (MAPs), important for maintaining axonal homeostasis [154]. Tau undergoes posttranslational modifications: phosphorylation and *O*-glycosylation [155]. The phosphorylation of tau is mediated by various protein kinases, and phosphorylation at serine 404 is mainly implicated in the loss of tau-mediated tubulin polymerization [156]. Dissociation of tau from microtubules followed by tubulin depolymerization occurs as a consequence of the phosphorylation of microtubule-associated tau (MAT) [157]. Furthermore, tau hyperphosphorylation forms insoluble fibers or aggregates termed paired helical filaments (PHF). PHFs further aggregate, leading to neurofibrillary tangles, which are one of the pathological hallmarks of AD [158].

LPA has been shown to induce tau phosphorylation and neurite retraction in an in vitro neuronal cell line. One of the important targets by which LPA induces tau phosphorylation is glycogen Synthase Kinase-3 β (GSK-3β). GSK-3β is an important regulatory enzyme implicated in various cellular processes and is highly expressed in the CNS [159]. Alterations in GSK-3β-regulated pathways have been shown to be associated with various diseases, including diabetes mellitus, AD, and cancer. Specifically, increased GSK-3β activity was noted in neuronal cell culture models of apoptosis and neurodegeneration and in in vivo models of focal ischemia [160]. Various proteins, such as metabolic proteins, cytoskeletal proteins, and transcription factors, are phosphorylated by GSK-3β [161]. More importantly, GSK-3β phosphorylates neuronal MAPs involved in microtubule stabilization [162,163,164]. LPA has been shown to increase GSK-3β activity, thereby increasing tau phosphorylation. One mechanism of LPA-mediated GSK-3β activation involves the Gα12/13-mediated RhoA/Rock pathway, as Rho GTPases are implicated in tau pathology. This represents an important link between microtubule and microfilament dynamics, which is altered in AD [165,166]. The RhoA/ROCK pathway has been shown to phosphorylate tau at various sites—for instance, at Thr245 and Ser409 [167]. Tau hyperphosphorylation induced by LPA occurs in two epitopes, i.e., PHF1 and AD-2. This tau hyperphosphorylation is blocked by the GSK-3 inhibitor lithium, confirming that GSK-3β is the serine–threonine kinase responsible for tau hyperphosphorylation. 

Neurite retraction is an important cellular process during neuronal development, especially during neurogenesis and neuritogenesis. Conversely, excess neurite retraction has been implicated in various pathological states such as neurodegeneration [168]. LPA-induced neurite retraction is mediated by tau phosphorylation, which involves an increase in GSK-3β and PKA activation. Additionally, p38 MAPK activation also induces neurite retraction [169]. During the development of the brain, the transient receptor potential channel, subfamily M, member 2 (TRPM2) regulates LPA-induced neurite retraction [170]. LPA treatment induced time-dependent activation of GSK-3β and associated neurite retraction in cerebellar granule neurons [168]; this indicates that these phenomena are not specific to a particular neuronal cell type and, instead, represent a widespread process in neuronal cells. GSK-3 activation by LPA in cerebellar granule neurons is mediated by an increase in tyrosine phosphorylation of GSK-3 isoforms α and β. In any case, neurite retraction cannot be completely blocked by inhibiting GSK-3, indicating that GSK-3 activation plays an important role but is not essential for neurite retraction, for which the involvement of other factors is needed. 

Post-mitotic hippocampal neurons undergo apoptosis and necrosis upon treatment with LPA; however, the molecular mechanisms have not been elucidated [80]. Moreover, GSK-3 activation in neuronal cells also leads to apoptosis [171]. Hence, LPA-induced GSK-3 activation could partially mediate the apoptotic response in mature neurons. LPA plays a significant role in tau hyperphosphorylation and PHF formation in AD brains. Consequently, determining the LPA levels in AD brains and age-matched control brains needs to be investigated. Besides, BBB dysfunction leads to increased permeability of LPA in the CNS, thereby increasing its levels in the injured brain. Traumatic brain injury, a major risk factor for AD, is associated with BBB dysfunction. Therefore, overexpression of LPA receptors with a subsequent increase in LPA responses (such as GSK-3 activation), occurs in neurons of AD patients. 

### 4.3. Other Effects

Various in vitro studies employing neuronal cells treated with LPA have shown decreased glutamate uptake in astrocytes and a sustained increase in neuronal intracellular calcium levels. In addition, neurite retraction and collapse of the axonal growth cone were noted [172,173]. Furthermore, disruption of the BBB due to alterations in the endothelial tight junctions were noted at high concentrations of LPA [174]. Stimulation of the Rho/Rho kinase (ROCK) signaling pathway and subsequent downstream expression of proteolytic enzymes, like matrix metalloproteinase 9 (MMP-9) and urokinase-type plasminogen activator (uPA) also induced BBB disruption by degrading the basement membrane and extracellular matrix [123]. LPA1 also plays an important role in adult hippocampal neurogenesis [175]. LPA1 is highly expressed in hippocampal progenitor cells, where it is involved in neural differentiation [176]; in comparison, LPA1 promotes synaptic alterations in adult hippocampal neurons [177]. Mice lacking LPA1 demonstrate hippocampal deficits associated with behavioral impairments, such as impaired spatial memory retention, altered exploration, and increased anxiety-like responses [178]. Furthermore, the role of LPA and its receptors in behavior and cognition has been validated by studies on LPA1-null mice, which have a targeted disruption in the *LPA1* gene. These mice show normal survival but display defective hippocampal neurogenesis, decreased brain-derived neurotrophic factor (BDNF) levels [179], and altered cortical development [175]. These mice also exhibit prepulse inhibition impairment and decreased ability to process irrelevant auditory stimulation, which contribute to the development of cognitive deficits. Behavioral studies showed impaired exploration in the open field (OF) and increased anxiety-like responses in the elevated plus maze (EPM) test. Additionally, impairment in spatial memory retention and abnormal use of searching strategies were noted [180,181]. Hence, LPA and its receptor mediate anxiety-like behavior and cognition deficits, especially spatial memory alterations, thereby indicating LPA role in major neuropsychiatric and cognitive disorders. The various effects of ATX–LPA signaling in Alzheimer’s disease are depicted in Figure 1.

## 5. Cross-Talk between ATX–LPA Signaling and Risk Factors of Alzheimer’s Disease

### 5.1. Traumatic Brain Injury and AD

Traumatic brain injury (TBI) is a significant health problem characterized by both acute and chronic disabilities; one significant long-term complication of TBI is neurodegeneration linked to AD. The distinctive pathologies of AD (Aβ and P-tau) are noted in the postmortem brains of TBI patients, indicating a strong association between neurodegeneration and TBI [182,183,184]. Autopsy findings show diffuse Aβ plaques in areas surrounding the lesion sites in TBI patients who died after an injury [185,186]. The mechanisms postulated for Aβ accumulation following TBI involve BBB disruption leading to ischemic insult [187]. Additionally, hypoperfusion and vascular dysfunction also contribute to Aβ deposition by increasing the activity of β- and γ-secretases [188,189]. Metabolic acidosis following TBI could also potentially contribute to Aβ accumulation, since Aβ, like other proteins, is prone to aggregation in a pH-dependent manner [190]. Similarly, mechanical stress following TBI disrupts the microtubule networks within the axons, leading to tau hyperphosphorylation [191]. Oxidative stress and cerebrovascular dysfunction also contribute to tau pathology. Hence, vascular damage and impairment following TBI lead to a chronic hyoperfusion state, which causes Aβ accumulation, tau hyperphoshorylation, and neuronal dysfunction, predisposing to AD. 

CNS injury is associated with increased concentrations of LPA in the brain and the CSF [120,192]. Following neurotrauma, induction of *LPA2* gene expression is observed in murine cortical astrocytes, spinal cord astrocytes, and human ependymal cells [192,193]. Similarly, in murine models, the expression of LPA1 is increased in spinal cord astrocytes while LPA3 expression is increased in cortical and spinal cord neurons. In the normal adult brain, the expression of LPA1–LPA3 is extremely low, but it becomes upregulated following brain injury [182]. Additionally, an increase in the levels of ATX was observed adjacent to injury lesions in rat cortical white matter [127], possibly suggesting possible overactive LPA signaling. Increase in the LPA pulse has been shown to occur in the early phase of a brain insult (i.e., within 4 h in the mouse model of controlled cortical impact (CCI) and within 24 h in TBI patients), indicating that LPA might be the first mediator responding to the injury. Increased LPA levels also enhance BBB permeability, thereby accounting for the hemorrhage and edema noted in TBI patients [122]. Each of these factors could potentially contribute to primary and secondary sequelae observed after TBI. Lastly, anti-LPA antibodies have been shown to improve patient outcomes following TBI [194]; this indicates the role of a dysfunctional ATX–LPA signaling, although the mechanistic validation of this association needs to be explored. The strong association between TBI and ATX–LPA signaling provides indirect evidence that dysfunctional ATX–LPA signaling can predispose to and contribute to the pathogenesis of AD; however, further studies are required to provide a direct, mechanistic evidence to understand the link between ATX–LPA and AD.

### 5.2. Metabolic Syndrome and AD

Various studies indicate a strong association between metabolic diseases, like obesity, diabetes mellitus, and dyslipidemia, and late-onset AD. Some of the alterations in the brain, including loss of brain volume and glucose hypometabolism, contribute to the pathology of AD [195,196,197]. Several factors contribute to the development of AD pathologies. For instance, metabolic disorders lead to chronic ischemia in the cerebral vasculature resulting in the degeneration of neurons. Insulin resistance, both peripheral and central (brain), leads to dysfunctional glucose metabolism in the neurons, thereby promoting oxidative stress, which causes neuronal insult in the brain [198,199,200]. Furthermore, these metabolic disorders are characterized by chronic inflammation, which alters BBB permeability and, over a period, leads to neuronal inflammation and degeneration [201]. 

An increase in the levels of pro-inflammatory cytokines and a downregulation of anti-inflammatory cytokines are noted in AD brains. Several lines of evidence indicate that dysfunctional ATX–LPA signaling predisposes to the pathologies of obesity and AD. ATX expression is upregulated in obese patients and mice models of obesity as a result of increased accumulation of triglycerides in the adipocytes [202,203]. This increased expression is also associated with insulin resistance and impaired glucose tolerance. Obese patients with diabetes or impaired glucose tolerance exhibited increased ATX expression compared to obese patients with normal glucose homeostasis [204]. Various inhibitors of ATX and LPA receptor antagonists are currently being investigated as possible treatments of obesity and associated metabolic disorders. 

Murine models injected with LPA showed defective insulin release from pancreatic β-cells in response to glucose administration. Interestingly, increased BBB permeability was noted, indicating that the effects of elevated ATX–LPA levels may be due to central and systemic factors [122]. Higher expression of the *ATX* gene was noted in the frontal cortex of AD patients compared to control brains [205]. Additionally, a study conducted by McLimans et al. showed significantly higher levels of ATX in mild cognitive impairment (MCI) and AD patients [206]. Increased ATX in AD was associated with decreased glucose uptake (hypometabolism), diminished performance on executive function and memory scores, and a reduced cortical thickness in the prefrontal cortex and medial temporal lobe. Finally, increased ATX showed a positive correlation with established metabolic and AD biomarkers, suggesting that ATX in the CSF could predict AD neuropathology similar to metabolic dysfunction. 

These results support the theory that ATX may be an indicator of central dysmetabolism for a variety of AD outcomes. Additional studies are necessary to determine if elevated ATX predicts AD and determines certain aspects of the disease. Future clinical and animal model studies should consider examining this biomarker and its applications as a potential target for pharmacologic therapies in the setting of glucose dysregulation and AD. Such work should also examine how ATX predicts changes in these outcomes over time.

### 5.3. Chronic Hypoperfusion and AD

Various studies have shown that cerebrovascular dysfunction (cerebral hypoperfusion, amyloid angiopathy of the capillaries, and BBB dysfunction) predisposes to AD [207,208,209]. Additionally, cerebrovascular dysfunction is implicated as an early event in AD pathogenesis. This leads to the speculation that hypoxia and ischemia enhance the pathogenesis of AD, as cerebral ischemia impairs the cognitive functions and aggravates A β-induced cognitive deficits [210,211,212]. The predominant cause of hypoxia is ischemia, which leads to the downregulation of synaptic transmission, neuroinflammation, and neuronal death [213]. LPA1 signaling and hypoxia are linked through diverse mechanisms. Hypoxia enhances LPA-induced hypoxia inducible factor-1 alpha (HIF-1α) expression and VEGF expression [214,215]. The presence of ATX is important for HIF-1α expression during the embryogenic period and is rescued by LPA exposure in ATX-null animals [216]. Short-term cortical exposure to hypoxia causes the overactivation of LPA1 and the downregulation of G protein-coupled receptor kinase 2 (GRK2) which can alter the normal brain development, resulting in various neurological and neuropsychiatric diseases [217]. Similar responses were noted upon stimulation of LPA1 signaling in NPCs. Additionally, these effects could be reduced or prevented by an LPA1–3 inhibitor, the use of LPA1-null mice [207], or the use of LPA1 and LPA2 double-null mice [64]. Hypoxia and HIF-1α are associated with the increase in the activities of β- and γ-secretases, whereas α-secretase activity is decreased in hypoxia. HIF-1α, by binding to the hypoxia-response element in the BACE1 promotor region, induces *BACE1* gene transcription [218]. Furthermore, the γ-secretase complex activity is enhanced by HIF-1α, though in a non-transcriptional manner [219]. In the brain, endoplasmic reticulum stress with associated reduction of autophagy is induced by ischemia and hypoxia. This leads to increased PS1 expression and activation of γ-secretase [220,221]. Finally, both PS1 and the APP intracellular domain peptide are known to induce HIF-1α protein [222,223].

Under hypoxic conditions, upregulation of LPA1 and LPA2 in the retinal layers following increased LPA signaling was noted, which promoted retinal cell survival [224]. However, conflicting results have been reported for retinopathy in a prematurity model of rat neonates induced by alternating hypoxic and hyperoxic conditions [225]. While a decrease in cell viability was noted with the overexpression of LPA1 or LPA exposure, LPA1 inhibition or short hairpin RNA knockdown showed protective effects on cell viability. These results indicate that there is a strong link between hypoxia/ischemia and LPAR signaling; however, LPA role as a deleterious or protective factor needs to be further investigated. 

## 6. Future Perspective on ATX–LPA Signaling

Recent research on the ATX–LPA signaling in the nervous system, especially in AD, has opened avenues for the strategic modulation of this signaling, thereby altering the natural history of the disease. ATX offers several advantages for developing therapeutic targets. For instance, its functions are mediated by the formation of an extracellular ligand (LPA) and, hence, the development of ATX inhibitors does not necessitate the formulation of a lipophilic compound to cross the BBB. Additionally, as an ecto-enzyme, it can be utilized in enzyme activity assays for drug optimization and characterization. The development of conditional ATX knockout mice along with LPA receptor-deficient mice could lead to the investigation of novel therapeutic compounds modulating ATX–LPA signaling in various disease states. 

The development of novel compounds targeting the ATX–LPA axis would be an attractive strategy and includes ATX inhibitors, LPA receptor antagonists, and LPA monoclonal antibodies. Some of these compounds are in various phases of clinical trials for cancer and chronic inflammation. For instance, a monoclonal antibody against LPA, named LpathomabTM, reduced IL-6 expression and lesion volume and improved the functional outcomes in a mouse model of traumatic brain injury and is currently in phase 1 clinical trials [226]. Other novel targets of the ATX–LPA signaling axis currently in phase II clinical trials for idiopathic pulmonary fibrosis are GLPG1690 (ATX inhibitor developed by Galapagos NV) and BMS-986020 (LPA1 antagonist). Sanofi SAR100842, an LPA1–3 antagonist, is in phase II trials for the treatment of systemic sclerosis [227]. Several additional compounds targeting ATX–LPA signaling are in preclinical studies for the treatment of various diseases.

The above-mentioned studies indicate that there is an enormous interest in studying the ATX–LPA signaling pathway by the scientific research community as well as the pharmaceutical industry to develop novel compounds to treat various diseases. As there are no clinically approved drugs targeting the ATX–LPA axis, future studies should test the potential of compounds targeting ATX–LPA signaling for the treatment of a wide range of neurological diseases, including AD.

## 7. Conclusions

Overwhelming evidence indicates that the ATX–LPA signaling axis plays key roles in the numerous processes central to AD by interacting with a series of LPARs. Moreover, the mechanisms involving the ATX–LPAR axis in AD pathogenesis have received adequate attention in recent years. Targeting the ATX–LPA pathway holds therapeutic potential because this signaling axis may promote the development of neurodegenerative diseases through multiple mechanisms involving mitochondrial dysfunction, oxidative stress, inflammation, and other processes. Elevated expression of LPA resulted from the activation of the ATX–LPARs axis. It has been reported that the levels of ATX are significantly increased in CSF and in the frontal cortex of AD patients. However, further studies must be conducted to analyze the aberrant expression of ATX and LPARs in AD. In addition, the mechanisms by which the ATX–LPA axis promotes AD development and progression are complex and not fully revealed; this demands intensive research on the signaling mechanisms. To summarize, ATX–LPA signaling plays an important role in the pathogenesis and progression of AD. The development of novel therapeutic compounds that inhibit ATX or block LPAR and its downstream signaling would be beneficial in the treatment of AD, in addition to ATX being a potential biomarker for AD.

## Figures and Tables

**Table 1:** Lysophosphatidic acid (LPA)-receptor mediated Gα signaling and its cellular effects.

Gα Subunit	Signaling Pathway	Cellular Effects	References
Gα12/13	Rho/ROCK and Rho/SRF pathways	Cell motility Invasion Cytoskeletal changes Vasodilation	[44,45,46]
Gαq/11	IP3-DAG pathway	Cell growth Immunity Learning and memory	[44,45,46,47]
Gαs	Adenylyl cyclase	Inhibits cell migration	[48]
Gαi/O	Ras/MAPK pathway PI3K/Rac pathway PI3K/Akt pathway	Reorganization of the actin cytoskeleton Cytoskeletal changes and cell migration Cell survival and apoptosis	[49,50,51,52]

**Table 2:** LPA receptor subtypes and their biological functions.

LPAR Subtypes	Biological Functions	References
LPA1	Cell survival, proliferation, adhesion, migration, immune function, and myelination	[53]
LPA2	Similar to and complementary to LPA1-mediated effects	[33,54]
LPA3	Mainly involved in reproductive functions—fertility, implantation of the embryo	[55]
LPA4	Cell adhesion and aggregation, vascular development, regulation of osteogenesis, offset LPA1- and LPA2-mediated chemokine release	[56,57]
LPA5	Inhibits cell motility Involved in chemokine release	[48,58]
LPA6	Not fully elucidated Mutations linked to hair loss and hypotrichosis	[59,60,61]

## Abbreviations

ATX	Autotaxin
AD	Alzheimer’s disease
BBB	Blood–brain barrier
BACE	β-site APP-cleaving enzyme
ENPP	Ecto-nucleotide pyrophosphatase/phosphodiesterase
GPCR	G protein-coupled receptor
GSK3β	Glycogen synthase kinase-3β
LPA	Lysophosphatidic acid
LPC	Lysophosphatidylcholine
